# What is the sensitivity and exactness of post-mortem diagnostic method for cardiopulmonary nematodes in wild carnivores? towards the gold standard

**DOI:** 10.1007/s11259-023-10084-3

**Published:** 2023-03-15

**Authors:** Irene Arcenillas-Hernández, M. R. Ruiz de Ybáñez, Carlos Martínez-Carrasco

**Affiliations:** Dpto. de Sanidad Animal, Facultad de Veterinaria, Campus de Excelencia Internacional “Campus Mare, Nostrum” Universidad de Murcia, Espinardo, Murcia, 30100 España

**Keywords:** Artificial digestion, Lungworms, Post-mortem diagnostic, Red fox

## Abstract

Cardiopulmonary nematodes cause health and fitness disorders in wild and domestic carnivores. The red fox (*Vulpes vulpes*) participates in the spread of these shared parasites at the domestic-wildlife interface. This study aimed to evaluate the sensitivity of post-mortem diagnostic method for detecting lungworms in carnivores, and its exactness to estimate the parasite intensity of each nematode species. Cardiorespiratory system of fifty-one foxes were examined through three consecutively methodological steps: first, the tracheobronchial tree, pulmonary arteries and their branches were opened (OT); next, lung parenchyma was immersed in water and squeezed (WS); finally, the parenchyma was artificially digested in a pepsin and chlorhydric acid solution (AD). *Eucoleus aerophilus*, *Angiostrongylus vasorum*, *Crenosoma vulpis* and *Metathelazia capsulata* were identified. The number of recovered nematodes in each step were 454 (OT), 285 (WS) and 141 (AD). The use of OT and WS helped to improve parasite intensity results and decreased false negative cases. Accordingly, when OT and WS were used together, the sensitivity in the detection of parasitized foxes was 96.1%, while the exactness of parasite intensity was 84%. When AD was performed, although sensitivity does not rise, results were more exact, increasing the total number of detected parasites by 16%. Moreover, AD improved the sensitivity in the detection of *A. vasorum* and *M. capsulata*, as well as quantifying more exactly the parasite intensity (92.5% and 92.3% of exactness without AD, respectively). Our study provides valuable information that should be taken into account when planning epidemiological studies based on cardiopulmonary nematode detection in carnivores.

## Introduction

Cardiopulmonary nematodes are common parasites of wild and domestic carnivores that inhabit mainly in the tracheobronchial tree (trachea, bronchi and bronchioles). It is widely recognized that lungworms cause respiratory diseases with serious health implications for the host (Traversa et al. 2010) and, consequently, may also influence wild population dynamics (Martínez-Rondán et al. 2019).

Environmental degradation of natural areas is one of the main factors why a large variety of wild species, including mammals, have expanded their home range into urban and peri-urban areas, attracted by the trophic resources available in anthropized environments. This leads to increased interaction among domestic animals, wildlife and humans (Plumer et al. 2014; Mackenstedt et al. 2015; Deplazes et al. 2019), promoting the spread of parasites to non-endemic areas and their establishment in domestic animals and, when possible, even in humans (Deplazes et al. 2004; Saeed et al. 2006; Traversa et al. 2010; Veronesi et al. 2014; Otranto et al. 2015).

Red fox (*Vulpes vulpes*) is one of the most widely distributed wild carnivore worldwide. This canid has great ecological plasticity, being capable of adapting to a wide range of habitats, including anthropized areas (Díaz-Ruiz et al. 2013; Hoffmann and Sillero-Zubiri 2016). Indeed, fox prefers fragmented and heterogeneous areas (Gloor et al. 2001). This wild carnivore is the definitive host of a wide variety of parasites, acting in some cases as a parasite reservoir (Deplazes et al. 2004; Reperant et al. 2007; Di Cerbo et al. 2008). Foxes share a large number of pathogens with other domestic canids, and even with humans, some of them being zoonotic parasites (Otranto and Deplazes 2019). Thus, the red fox is a relevant species from an epidemiological point of view at the domestic-wildlife-human interface, due to the possibility of transmission of these pathogens (Dáttilo et al. 2020; Tayyrov et al. 2021). Considering this role, a risk exists in those shared territories where an interaction between domestic, anthropic and sylvatic life cycles of these parasites may occur (Traversa et al. 2010).

*Eucoleus aerophilus*, *Crenosoma vulpis* and *Angiostrongylus vasorum* are lungworms previously described in wild and domestic canids worldwide (Morgan et al. 2005; Traversa et al. 2010; Tolnai et al. 2015; Deak et al. 2020). Specifically, *E. aerophilus* (syn. *Capillaria aerophila*) is located on the tracheal and bronchial mucosa of a broad range of canids, felids, mustelids and even humans (Anderson 2000; Lalošević et al. 2008; Traversa et al. 2009; Di Cesare et al. 2014; Tolnai et al. 2015), whereas *C. vulpis* infects the bronchioles, bronchi and trachea of domestic and wild canids (Anderson 2000; Schug et al. 2018; Deak et al. 2020; Pohly et al. 2022) and mustelids (Anderson 2000; Akdesir et al. 2018; Figueiredo et al. 2018) and ursids such as brown bear (*Ursus arctos*) or American bear (*Ursus americanus*) (Rogers and Rogers 1976; Borka-Vitalis et al. 2017; Mahjoub et al. 2020). Both nematodes can cause mucosal oedema and lung disorders (Nevárez et al. 2005). On the other hand, *A. vasorum* is a nematode of the pulmonary arteries and the right side of the heart that can produce progressive deterioration of the respiratory and cardiac functions or even neurological disorders (Alho et al. 2018; Carretón et al. 2020). Nowadays this nematode species is considered an emerging lungworm in Europe both in domestic and in wild canids (Helm et al. 2010; Deak et al. 2017; Tayyrov et al. 2021; Tieri et al. 2021). Apart from the red fox, other definitive host of A. *vasorum* are wild carnivores such as the grey wolf (*Canis lupus*) (Segovia et al. 2001), Eurasian badger (*Meles meles*) (Torres et al. 2001), otter (*Lutra lutra*) (Madsen et al. 1999), coyote (*Canis latrans*) (Priest et al. 2018) and the recently detected golden jackal (*Canis aureus*) (Gavrilović et al. 2019).

All these nematodes have been previously described in foxes from Iberian Peninsula (Martínez-Carrasco et al. 2007; Garrido-Castañé et al. 2015; Martínez-Rondán et al. 2019; Carretón et al. 2020). Other cardiopulmonary species described in red fox are the heartworm *Dirofilaria immitis* (Gortázar et al. 1998; Segovia et al. 2004), *Filaroides hirti* (Martínez-Rondán et al. 2019) and *Metathelazia capsulata*, a poorly described nematode found in the bronchi of foxes and mustelids (Jiménez et al. 2013; Arcenillas-Hernández et al. 2023).

Red fox is thought to be a key reservoir of cardiopulmonary nematodes worldwide, being one of the main drivers of the increase of cases in dogs (Traversa et al. 2010; Veronesi et al. 2014). The emergence of cardiopulmonary parasites in pets has led to a growing interest to understand the causes of their occurrence (Deak et al. 2020; Fuehrer et al. 2021). Therefore, it is necessary to carry out epidemiological studies based on the diagnosis of cardiopulmonary nematodes in pets and also in foxes, especially in those areas where the interaction between these host species is more intense (De Zan et al. 2021).

Many of the studies published to date aimed at detecting the presence and parasite intensity of cardiopulmonary nematodes in carnivores are based on the isolation of these parasites by necropsy. However, to the authors’ knowledge, none of them have evaluated the sensitivity of this diagnostic method. Assessing the effectiveness of the method applied for epidemiological studies is fundamental, since the choice of the most appropriate method guarantees the most accurate results and can also reduce the costs and/or the execution time of the study (Llewellyn et al. 2016; Buonfrate et al. 2018). The most frequently used diagnostic method for detecting cardiopulmonary nematodes in carnivores is the opening of the tracheobronchial tree, heart chambers and the pulmonary arteries and their branches. We propose the evaluation of the sensitivity of this method in the detection of parasitized red foxes and the precision in quantifying the parasite intensity of each cardiopulmonary nematode species. Specifically, we evaluated the accuracy of the subsequent application of two consecutive steps: first, the immersion in water and squeezing of the lung parenchyma and, second, the artificial digestion of lung tissue. The improvement of this diagnostic method could be particularly significant to address studies in areas where wild and domestic carnivores share the same habitat, with a high risk of transmission of lungworms.

## Materials and methods

### Study samples

Between 2015 and 2018, 51 red foxes (23 females and 28 males; 17 juveniles and 34 adults) shot in authorized hunts or found dead by roadkill in Murcia and Alicante provinces (SE Spain) were necropsied. The sex and age of the animals were recorded, as well as their weight were divided into three categories: low (< 5 kg), medium (5–7 kg) and high (> 7 kg). The age category was determined by tooth growth (Harris 1978). Heart and lungs, including the trachea, were extracted, individually refrigerated in plastic labelled bags and submitted to the laboratory, where they were frozen at -20ºC until their analysis.

### Laboratory procedures

Analysis of the cardiopulmonary system included three consecutive steps: first, the heart chambers, the tracheobronchial tree and the pulmonary arteries and their branches were opened (OT); next, the lung parenchyma was immersed in water and squeezed (WS); finally, the lung tissue was artificially digested in a solution of pepsin and chlorhydric acid (AD). Briefly, after defrosting the samples at laboratory temperature, the tracheobronchial tree and the large pulmonary vessel were longitudinally opened and inspected under the stereoscope to isolate the nematodes. Also, the heart was separated from the lungs as close as possible to the main vessels, and chambers were opened and inspected. To conclude the first step of the analysis (OT), trachea and cardiac chambers were washed and filtered through a 63 μm of mesh sieve, and the retained material was examined under the stereomicroscope.

For washing and squeezing the respiratory tracts (WS), lung pieces (3 × 3 cm) were immersed in a bucket with water and manually squeezed for one minute. Subsequently, the water was filtered as previously described, and the retained material was deposited in a Petri dish and examined under a stereomicroscope to remove all nematodes located in small vessels and bronchi which were not detected with the previous step (OT).

Finally, all the pieces of lung parenchyma were artificially digested (AD) as described in Martínez-Rondán et al. (2019) with some modifications. Specifically, the tissue was digested in a solution with pepsin and chlorhydric acid (1.5%) in distilled water, under constant and slight shaking, at 40ºC for 15 min. After this time, the digestion sediment was recovered and examined under the stereomicroscope. When parts of the parenchyma remained undigested, this procedure was repeated as described above.

The nematodes isolated in each methodological step (OT, WS or AD) were cleaned in distilled water and separately preserved in 70% ethanol until their morphometric identification based on Anderson et al. (2009), Costa et al. (2003), Jiménez et al. (2013) and Latrofa et al. (2015).

### Statistical analysis

Prevalence (P) and median intensity (MI) were calculated following Margolis et al. (1982) and Bush et al. (1997). According to quartiles, four categories were used to evaluate overall parasite intensity (very low: 1–2, low: 3–5, medium: 6–20 and high: >20 nematodes), and three categories (low, medium and high) for each nematode species identified (*E. aerophilus*: 1–2, 3–4, > 4 specimens; *A. vasorum*: 1–5, 6–20, > 20 specimens; *C. vulpis*: 1, 2, > 2 specimens; and *M. capsulata*: 1–3, 4–8, > 8 specimens). The distribution of data was analysed with Shapiro-Wilks test. Non-parametric Kruskal-Wallis test and Yate’s-corrected chi-squared test were used to compare medians and proportions, respectively. R software 4.1.2 (R Core Team 2021) software was used and significance threshold was established for the value p ≤ 0.05.

The sensitivity of a diagnostic method is defined by the formula: TP/(TP + FN), where TF is a true positive result and FN is a false negative result. Other words, it is the ability of the method to accurately classify infected animals (Rutjes et al. 2007; Carrau et al. 2021). In our study, TP occurred when cardiopulmonary nematodes were isolated in the studied fox, while FN corresponded to animals in which these parasites were not detected despite being present in the cardiorespiratory system of the host. Specificity is defined by the formula: TN/(FP + TN), where TN is a true negative result, and it is referring to the capacity of the method to detect not parasitized individuals (Coughlin et al. 1992). In this study, the specificity of the method was not taken into account, since the use of AD ensures the identification of 100% of negative foxes (i.e., not parasitized by cardiopulmonary nematodes). Moreover, the identification of the four nematode species found is certain on the basis of the taxonomic keys used, with no possibility of confusion with other nematode species.

## Results

Overall, 880 cardiopulmonary nematodes were isolated from the samples, and four species were identified: *E. aerophilus*, *A. vasorum*, *C. vulpis* and *M. capsulata*. The highest number of nematodes were detected by OT (454/880, 51.6%, MI 4 specimens, range 1–70). In the next methodological step (WS), another 285 nematodes were obtained (285/880, 32.4%; MI 4.5; range 1–35), while in the last step (AD), 16.0% of the total number of isolated nematodes were detected (141/880, MI 2, range 1–20) (Table 1). Attending to nematode species, 100% of *E. aerophilus* and 98.4% of *C. vulpis* were collected when the first two steps (OT and WS) were applied. In the case of *A. vasorum* and *M. capsulata*, the percentage of recovery by the first two steps was lower (84.4% and 76.6%, respectively), being necessary to digest the lung parenchyma (AD) to detect the remaining specimens of each species (15.6% and 23.4%, respectively) (Table 1).

**Table 1:** Distribution of the number (N) and percentage of specimens (P), median intensity (MI) and range of each nematode species isolated from red foxes by opening (OT), squeezing (WS) and artificial digestion (AD).

	OT		WS		AD		
Nematodes	N (%)	MI (range)	N (%)	MI (range)	N (%)	MI (range)	Total specimens
*E. aerophilus*	81 (97.6)	2 (1–15)	2 (2.4)	1 (1–1)	0	0	83
* A. vasorum*	200 (48.6)	3.5 (1–65)	147 (35.8)	6 (1–35)	64 (15.6)	3 (1–14)	411
* C. vulpis*	48 (78.7)	1 (1–21)	12 (19.7)	4 (1–7)	1 (1.6)	1	61
*M. capsulata*	125 (38.5)	2 (1–70)	124 (38.1)	2 (1–27)	76 (23.4)	2 (1–20)	325
All species	454 (51.6)	4 (1–70)	285 (32.4)	4.5 (1–35)	141 (16)	2 (1–20)	880

Overall, the sensitivity of detection of cardiopulmonary nematodes was 80.4% (41/51) when OT was the only procedure used, while it increased to 96.1% (49/51) when OT and subsequent WS were applied. Finally, when the three steps (OT + WS + AD) were used, sensitivity increased by 3.9% (2/51).

According to each of the nematode species found, a sensitivity of 100% was obtained when using OT and subsequently WS in the case of *E. aerophilus* and *C. vulpis* (Table 2). When only OT was used, the number of false negatives was high for *A. vasorum* (30.8%) and *M. capsulata* (51.9%), increasing the sensitivity to 92.3% and 92.6%, respectively, when the second methodological step (WS) was performed. It is relevant to note that up to 7.7% of foxes infected with *A. vasorum* and 7.4% of those harbouring *M. capsulata* would not have been detected as carriers of these nematode species if the last step of the diagnostic method (AD) had not been used.

**Table 2:** Sensitivity of the detection of the four cardiopulmonary nematode species described in the study using the first two consecutive steps diagnostic method: opening (OT) and squeezing (WS), but without artificial digestion

	OT				OT + WS			
Nematodes	TP^(a)^	FN^(b)^	Sensitivity (%)	%FN	TP^(a)^	FN^(b)^	Sensitivity (%)	%FN
*E. aerophilus*	26	1	96.3%	3.7%	27	0	100%	0%
*A. vasorum*	18	8	69.2%	30.8%	24	2	92.3%	7.7%
*C. vulpis*	8	1	88.8%	11.2%	9	0	100%	0%
*M. capsulata*	13	14	48.1%	51.9%	25	2	92.6%	7.4%

(a) True positive foxes; (b) False negative foxes

The evaluation of biotic factors (age, sex and weight of the foxes) did not show a significant relationship with either prevalence or parasite intensity in spite of the use of each methodological step or even the whole procedure (p-value > 0.05) (Table 3).

**Table 3:** Number of parasitized foxes (P: prevalence) and intensity of nematodes (MI: median intensity) detected by the three-step diagnostic method (opening: OT; squeezing: WS; artificial digestion: AD) attending to the sex, age and weight of the host

			Diagnostic procedure					
			OT		OT + WS		OT + WS + AD	
Variable	Level	Number of foxes	P (%)	Intensity (MI)	P (%)	Intensity (MI)	P (%)	Intensity (MI)
Sex	Male	28	85.7%	299 (5)	100%	475 (7)	100%	566 (9.5)
	Female	23	73.9%	155 (2)	91.3%	264 (3)	100%	314 (3)
Age	Juvenile	17	88.2%	150 (4)	94.1%	251 (7.5)	100%	295 (8)
	Adult	34	76.5%	304 (3)	97%	488 (4)	100%	582 (5)
Weight	Low	13	92.3%	110 (3.5)	100%	187 (4)	100%	208 (4)
	Medium	25	80%	207 (3.5)	92%	336 (7)	100%	427 (9)
	High	13	69.2%	137 (4)	100%	216 (3)	100%	245 (3)

The use of OT as the unique methodological step resulted in the detection of only 56.2% of the positive animals with an overall very low parasite intensity (1–2 cardiopulmonary nematodes), showing statistically significant differences (p-value < 0.05) with the results of foxes with any of the remaining ranges of parasite intensity: 90.9% (low parasite intensity), 83.3% (medium parasite intensity) and 100% (high parasite intensity) (Table 4). In addition, in the case of foxes with an overall very low parasite intensity, 12.5% were false negatives when OT and WS were applied consecutively, being only confirmed as true positives when AD was used. Although differences were not statistically significant, the number of nematodes isolated increased when AD was included in the diagnostic procedure, improving the exactness. Specifically, 20% (4 nematodes), 15% (6 nematodes), 22.2% (32 nematodes) and 14.6% (99 nematodes) of the total number of nematodes isolated from foxes with very low, low, medium and high parasite intensity, respectively, were detected when using AD. In this sense, the exactness improves when, at least, OT and WS are used as a two-step method of parasite detection as is shown in Table 4.

**Table 4:** Positive foxes (P (%)) and number of nematode specimens (n) detected by the three-step diagnostic method (opening: OT; squeezing: WS; artificial digestion: AD) attending to the global parasite intensity ranges

			Diagnostic procedure					
		OT		OT + WS		OT + WS + AD		
Intensity range	Number of foxes	P (%)	n	P (%)	n	P (%)	n	Exactness (OT + WS)
Very low (1–2)	16	56.2% ^(*)^	10	87.5%	16	100%	20	80%
Low (3–5)	11	90.9%	22	100%	34	100%	40	85%
Medium (6–20)	12	83.3%	51	100%	112	100%	144	77.7%
High (> 20)	12	100%	371	100%	577	100%	676	85.3%
Total specimens			454		739		880	83.9%

^(*)^ Statistically significant differences (p-value < 0.05)

The lowest sensitivity of detection by OT of *M. capsulata* and *A. vasorum* was recorded in the groups of foxes with low parasite intensity, showing 68.8% and 42.6% of false negatives (Table 5), although differences were only statistically significant for *M. capsulata* (p < 0.05). This percentage remains remarkable for both nematode species (12.5% and 14.3% of false negatives, respectively) when the diagnostic procedure included two steps (OT followed by WS). Interestingly, the use of the OT step detected 100% of the foxes with a high parasite intensity of each of the four species of cardiopulmonary nematodes found (Table 5).

**Table 5:** Number of parasitized foxes (P: prevalence) and number of nematode specimens (ns) detected by the three-step diagnostic method (opening: OT; squeezing: WS; artificial digestion: AD) attending to the parasite intensity ranges of each cardiopulmonary nematode species

			Diagnostic procedure		
			OT	OT + WS	OT + WS + AD
Species	Intensity range	Number of foxes	P (%) (ns)		
*E. aerophilus*	1–2	17	94.1% (21)	100% (22)	100% (22)
	3–4	5	100% (17)	100% (17)	100% (17)
	> 4	5	100% (43)	100% (44)	100% (44)
*A. vasorum*	1–5	14	57.4% (11)	85.7% (21)	100% (28)
	6–20	6	66.6% (22)	100% (56)	100% (74)
	> 20	6	100% (167)	100% (270)	100% (309)
*C. vulpis*	1	6	83.3% (5)	100% (6)	100% (6)
	2	1	100% (2)	100% (2)	100% (2)
	> 2	2	100% (41)	100% (52)	100% (53)
*M. capsulata*	1–3	16	31.2% ^(*)^ (6)	87.5% (9)	100% (25)
	4–8	5	40% (3)	100% (23)	100% (30)
	> 8	6	100% (116)	100% (207)	100% (270)

^(*)^ Statistically significant differences (p-value < 0.05)

Finally, the implementation of AD as the last step allowed the detection of specimens of *A. vasorum* and *M. capsulata* that were not found with the two previously applied steps (OT and WS). Specifically, in the case of *A. vasorum* it was possible to detect 25% of the total nematodes (7/28 nematodes) in the category of foxes with low parasite intensity, 24.3% (18/74) in foxes with medium parasite intensity, and 12.6% (39/309) in animals with high parasite intensity. In the case of *M. capsulata*, these same parasite intensity categories were 24% (6/25), 23.4% (7/30) and 23.4% (63/270), respectively (Table 5).

## Discussion

To the authors’ knowledge, to date no study evaluated the sensitivity and exactness of the commonly used diagnostic method for the detection and quantification of cardiopulmonary nematodes in carnivores. This method consists on opening the tracheobronchial tree, the heart chambers, and the pulmonary arteries and their branches. To know the sensitivity of this method, it is necessary to have a gold standard procedure that allows knowing exactly the number of nematodes in the cardiorespiratory system. In our study, we have applied a procedure based on three consecutive steps (OT, WS, AD) that, together, allow all nematodes to be detected and, therefore, should be considered the gold standard method. Three of the cardiopulmonary nematode species detected (*E. aerophilus*, *C. vulpis* and *A. vasorum*), had been previously described parasitizing foxes in the Iberian Peninsula (Martínez-Carrasco et al. 2007; Garrido-Castañé et al. 2015; Fanelli et al. 2019; Martínez-Rondán et al. 2019). However, the fourth recovered species, *M. capsulata*, has not been recently recorded worldwide. The last reported description of this species in foxes was in Israel in the middle of the last century (Gerichter 1948), and later the nematode was only cited in badgers (Pence and Dowler 1979). Recently, a new species of this same genus, *M. mexicana*, has been described in nine-banded armadillo (*Dasypus novemcinctus*) from Central Mexico (Jiménez et al. 2013). To the author’s knowledge, this is the first report of this nematode species in red foxes from Iberian Peninsula (Arcenillas-Hernández et al. 2023).

Since the application of the three diagnostic steps was performed consecutively, the first one (OT) was the step with the highest recovery of nematodes as expected, followed by WS and, finally, AD. The use of OT and subsequently WS allowed the detection of a high percentage of parasitized animals (96.1%). In terms of overall prevalence of cardiopulmonary nematodes, our results show that if AD is not applied as the last step in lung processing, the method will have a 3.9% false negative rate. These results, although represent a small percentage, should be taken into account when conducting epidemiological studies based on OT and WS exclusively. Regarding the parasite intensity estimation, our study shows that the exactness of the method is reduced when AD is not used (84% when OT and WS were applied), especially in the quantification of *A. vasorum* and *M. capsulata* (84.4% and 76.6%, respectively). In the case of *E. aerophilus* and *C. vulpis*, exactness values were higher although AD were not used (100% and 98.3%, respectively). Globally, when AD was used, 141 more nematodes were detected (16%). In this regard, our results indicate that better records were obtained when two consecutive 15 min-AD steps were performed instead of only one lasting 30 min. This difference helped to prevent nematodes to be digested.

Most of the studies focused on the estimation of lungworms in foxes only use opening and/or flushing (Jeffery et al. 2004; Santoro et al. 2015; Houpin et al. 2016; Deak et al. 2020; Gillis-Germitsch et al. 2020; Lemming et al. 2020). However, results are not always comparable since the method used in these studies is not uniform in all cases (Lemming et al. 2020). Moreover, the aim of many studies is to determine the presence/absence of cardiorespiratory nematodes, without evaluating in many cases the parasite intensity (Schug et al. 2018). This highlights the need to standardize the diagnostic method for parasites in carnivores based on the necropsy of the cardiorespiratory system.

There are no similar studies evaluating the sensitivity and exactness of cardiopulmonary nematode detection using different methods. Only Houpin et al. (2016) compared the results of three methods for the detection of *A. vasorum*: dissection and visual examination (comparable to OT), PCR and a canine antigen detection ELISA test). The sensitivity of detection of *A. vasorum* using the OT method described by these authors reached 84.1%, while in our study it was 69.2%. However, the application of the second step (WS) in our study reached a sensitivity of 92.3%.

Sensitivity of detection and exactness of the parasite intensity quantification found by our three-step method varied attending to the lungworm species. Specifically, the sensitivity was 100% for detecting *E. aerophilus* and *C. vulpis* infected foxes, and the exactness of the parasite intensity was 100% and 98.4%, respectively, when OT and subsequently WS were used. These results are consistent with the fact that *E. aerophilus* is usually detected in the upper parts of the respiratory tract (trachea and large bronchi) and *C. vulpis* is localized in bronchi and bronchioles (Nevárez et al. 2005), so the application of AD does not represent a significant step to extract these nematode species from their usual location.

On the other hand, sensitivity of detection of positive foxes to *A. vasorum* and *M. capsulata* was below 93% when AD step was not used, meaning that 7.7% and 7.4% of parasitized foxes by these nematode species were false negative, respectively. In terms of intensity, 15.6% of *A. vasorum* specimens parasitizing foxes (range: 1–14) were found using AD. This is justified by the location of these nematodes in areas of the lung that make their detection by OT and WS difficult. In this sense, adults of *A. vasorum* are usually located in the right ventricle and major pulmonary arteries, but also in capillary vessels (Carretón et al. 2020; Morchón et al. 2021), showing a great difficulty to be opened, even using fine-tipped scissors. So, artificial digestion represents a necessary step to extract the nematodes from these parts of the lung parenchyma. *A. vasorum* is highly pathogenic to domestic canids (Poli et al. 1991; Morgan et al. 2008). In fact, high-loaded *A. vasorum* lungs can be easily recognized as they are much heavier and denser than healthy ones, showing yellow calcareous deposits that denote the existence of damaged areas (Jeffery et al. 2004). Therefore, when a high intensity of *A. vasorum* can be perceived visually, the use of the whole procedure could be recommended to provide more exact parasite intensity results.

Likewise, *M. capsulata* collected by AD represented 23.4% (range: 1–20) of the total number of these nematodes isolated in this study. This species is found mainly in bronchi and bronchioles (Gerichter 1948; Pence and Dowler 1979) and, as previously mentioned, artificial digestion allows a better isolation of specimens located in bronchioles of smaller diameter.

As noted above, the small capillaries or bronchioles are not often examined in detail when using only OT, either with or without the later use of WS, so it is not possible to recover all the nematodes present in the host nor to detect all the positive animals. Consequently, prevalence, abundance or parasite intensity rates could be underestimated (Morgan et al. 2008). Particularly, our results showed that up to 25% and 24% of the specimens of *A. vasorum* and *M. capsulata* would not be detected if AD step was not included in the diagnostic evaluation of foxes with low parasite intensities. Therefore, AD is necessary to improve the recovery of nematodes as well as to decrease false negative cases (Martínez-Rondán et al. 2019).

The use of the WS step in addition to the classical OT step is decisive in estimating the prevalence of parasitized foxes with a low intensity of *M. capsulata*, since the number of positive animals detected with these two phases is significantly higher than that described in the case of exclusively using OT.

Sex, age, and weight of foxes in no case influenced the results of the diagnosis of cardiopulmonary nematodes, which shows that the three-step method we describe can be applied in a general way in carnivores, regardless of the category to which the host belongs.

According to the prevalence of parasitized foxes registered by using the first two steps of the proposed method (OT and WS), the use of a costly and time-consuming technique such as AD should be carefully evaluated since it will only increase the number of positive animals by 3.9%. On the other hand, AD should be of great interest if the objective of the study is to quantify the parasite intensity on the host, obtaining the exact number of specimens.

The results achieved in the present study allow us to assure that the classic method used so far to detect the presence of cardiopulmonary nematodes in carnivores, only based on the opening of the airways and the heart together with washing and squeezing of these tissues, is not sufficient to precisely analyse small respiratory organs as well as to detect the presence of very small nematodes. In these circumstances, the addition of a supplementary diagnostic step, the artificial digestion of lung tissue, should be convenient. Furthermore, this three-step method has proved to be of particular interest in heavily parasitized animals. The fox is a synanthropic carnivore species that shares habitat with other domestic carnivores (mainly dogs and cats) and even humans. Their faeces, which may contain infective eggs or larvae, could be a source of infection for stray and domestic dogs (Traversa et al. 2014), and even humans could be affected with several bronchopulmonary zoonotic nematodes such as *E. aerophilus* (Otranto and Deplazes 2019). The diagnosis of cardiopulmonary nematodes in wild carnivores can be made by means of coprological techniques (Lalošević et al. 2013; Lempereur et al. 2016), larvae detection by the Baermann method (Morandi et al. 2019) or histopathological examination (De Liberato et al. 2017), supplying valuable information for the epidemiological study of these parasites and to know the degree of lung damage they can cause. However, detecting the presence of adult nematodes by means of a complete examination of the cardiopulmonary system allows obtaining more precise information that cannot be provided by the aforementioned techniques. Specifically, the implementation of the method described in the present study could bring precise information on the distribution of cardiopulmonary nematodes on wild carnivores, nowadays submitted to deep investigation given the possibility that domestic animals acquire these infections by contact with wild carnivores, even more in the present circumstances, in which the approach of populations of wild carnivores to anthropized areas has been widely verified. This knowledge would allow to significantly improve the design of the wildlife management plans currently in force.

## Data Availability

The datasets generated during and/or analysed during the current study are available from the corresponding author on reasonable request.
